# Traumatic Testicular Dislocation Complicated by Torsion Post Hernioplasty: A Case Report

**DOI:** 10.7759/cureus.86057

**Published:** 2025-06-15

**Authors:** Robyn Cabral, David M Milne, Keegan Dos Santos, Raegine Lovell, Shastri Sookhai, Shamir O Cawich

**Affiliations:** 1 General Surgery, Port of Spain General Hospital, Port of Spain, TTO; 2 Faculty of Medical Sciences, The University of the West Indies, St. Augustine, TTO

**Keywords:** hernioplasty complications, testicular ischemia, testicular torsion, testicular trauma, traumatic testicular dislocation

## Abstract

Testicular ischemia and atrophy are rare complications associated with inguinal hernia repair. Atrophy of the testicle can result in psychological distress along with infertility. This complication of inguinal hernia repair is associated with fibrosis of the testicular vasculature, resulting from the mesh used during routine hernia repair surgery. A 56-year-old male presented acutely with a tender left iliac fossa mass and pain. The patient gave a history of left inguinal hernia repair 10 years prior, which had proceeded without complication. Of note, the patient recounted an incident of testicular trauma in his early life, before any surgical intervention, after which he had experienced inability to palpate a left testicle. Upon acute presentation, the patient was clinically diagnosed with a recurrent left inguinal hernia with incarceration. He was promptly taken to the operating theatre. However, intraoperatively, instead of herniated intra-abdominal content, a torted, atrophic left testicle was noted at the superficial inguinal ring. Another rare testicular complication that presented simultaneously in this case was traumatic testicular dislocation (TTD). This condition is associated most often with blunt scrotal trauma and results in malposition of the testes out of the scrotum. We discuss a rare case of concomitant presentation of TTD, complicated by testicular atrophy and torsion in a single patient. A review of the relevant literature revealed that testicular atrophy, a rare complication of testicular ischemia, can result from the use of mesh in hernioplasty.

## Introduction

Testicular atrophy is a rare complication of inguinal hernia repair, and it results from injury to vessels traversing the inguinal canal [1]. It has an incidence rate of approximately 0.5% [2]. While not the sole precipitant of testicular ischemia, the use of mesh in hernioplasty has been implicated as a notable cause. Mesh causes a reactive fibrosis, which can affect the testicular vasculature, resulting in impairment of blood flow. More commonly, testicular atrophy results from ischemia to the testis by way of thrombosis of venous outflow rather than a deficit of arterial supply. Traumatic testicular dislocation (TTD) is another rare complication of the testicle. This condition results from trauma to the scrotum, with most cases associated with blunt trauma. A study by Chiu et al. [2] revealed large numbers of TTD associated with motorcycle accidents. In such cases of polytrauma, a diagnosis of TTD can be overlooked, masked by more obvious life-threatening injuries, such as haemorrhage, fracture of the pelvis and long bones [3]. We discuss an unfortunate case of TTD compounded by testicular atrophy. These two conditions presented simultaneously, despite varying etiologies due to distinct incidents that had occurred years apart.

## Case presentation

A 56-year-old male presented with a one-year history of intermittent pain in the left iliac fossa. One week preceding the presentation, he had reported exacerbation of left iliac fossa pain with swelling, the onset of which he associated with strenuous construction activities. Two days before the presentation, the pain had worsened, leading him to seek medical attention. Notably, 10 years prior, a left inguinal Lichtenstein tension-free hernia repair had been performed using polypropylene mesh, without any incident or unusual findings.

Upon current presentation, all blood investigations and vital signs were within normal limits. On examination, the patient’s abdomen was soft and non-tender, with swelling to the left iliac fossa noted. The swelling was tender to palpation and irreducible. There was a scar indicative of the previous hernia repair, with no overlying skin change and no cough impulse present. Upon examination of the scrotum, only the right testicle was palpable. When queried, the patient recalled the presence of bilateral testicles in childhood but reported a blow to his scrotum in his formative years, over 40 years prior, after which he could no longer palpate a left testicle. In his adult life, he had sought medical advice regarding the missing testicle, which had led to an investigation via ultrasonography, revealing nil evidence of the left testicle. The accuracy of this report could not be verified as it was only verbally obtained from the patient, and the ultrasound report was not seen. The patient had accepted the ultrasound report findings, and no further investigation regarding this had been performed.

**Figure 1:**
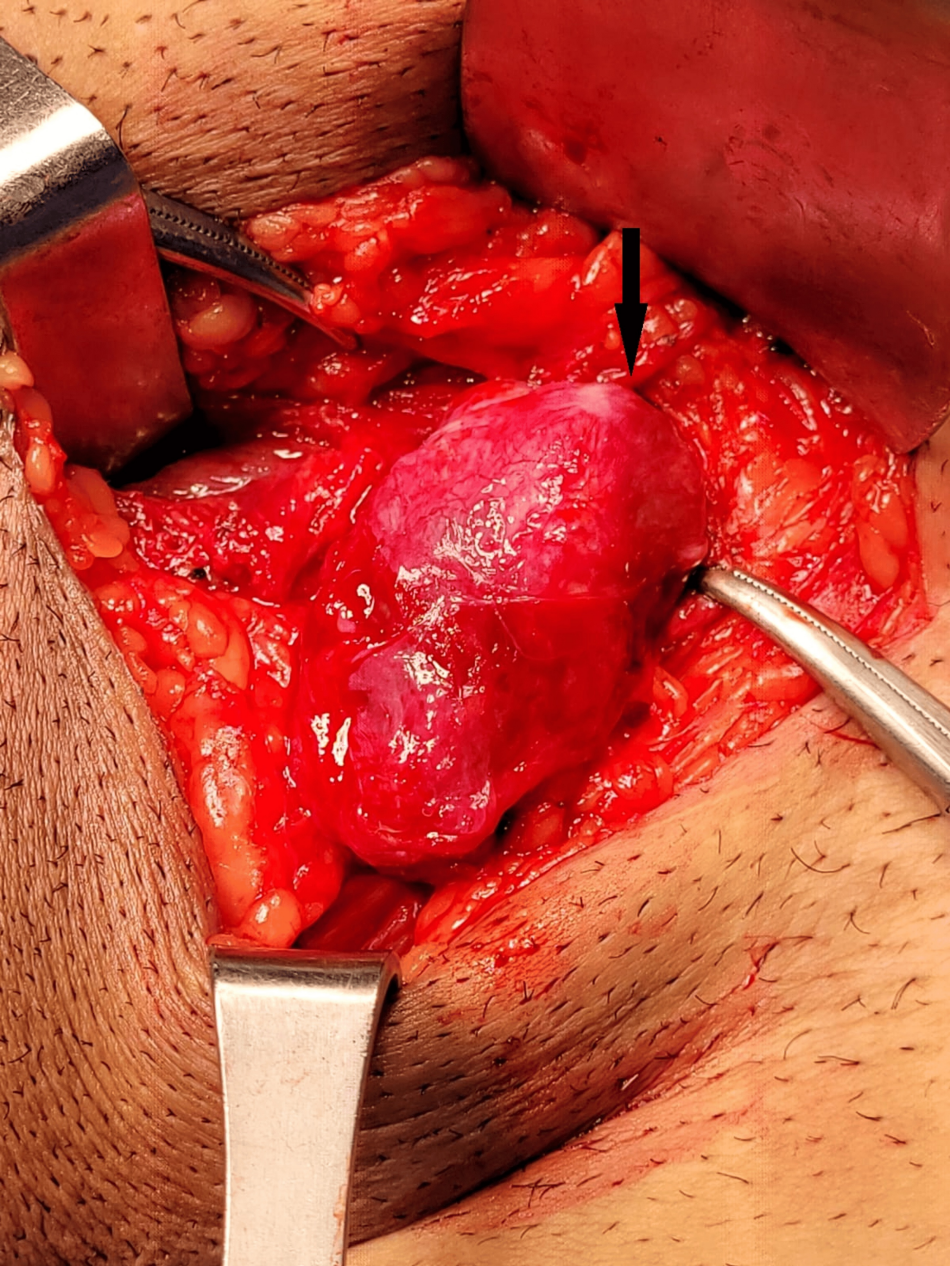
Left groin incision revealing atrophied left torted testicle at left superficial inguinal ring The black arrow illustrates the left torted testicle

In the emergency department, a clinical diagnosis of strangulated recurrent left inguinal hernia was made, and the patient was promptly taken to the operating theatre. A rapid sequence induction was performed, and the patient was cleaned and draped. An incision was made in the left iliac fossa, excising the previous scar, and the abdomen was opened in layers until the external oblique aponeurosis was reached. The superficial ring was located. A dusky mass approximately 3 x 3 x 2 cm was identified protruding from the superficial ring (Figure 1). No spermatic cord structures were palpable or visible upon initial observation, as the mass snugly plugged the superficial inguinal ring. The external oblique aponeurosis was then opened, and the mass was noted to be attached to 1 cm of cord structures, which had entered the deep ring.

**Figure 2:**
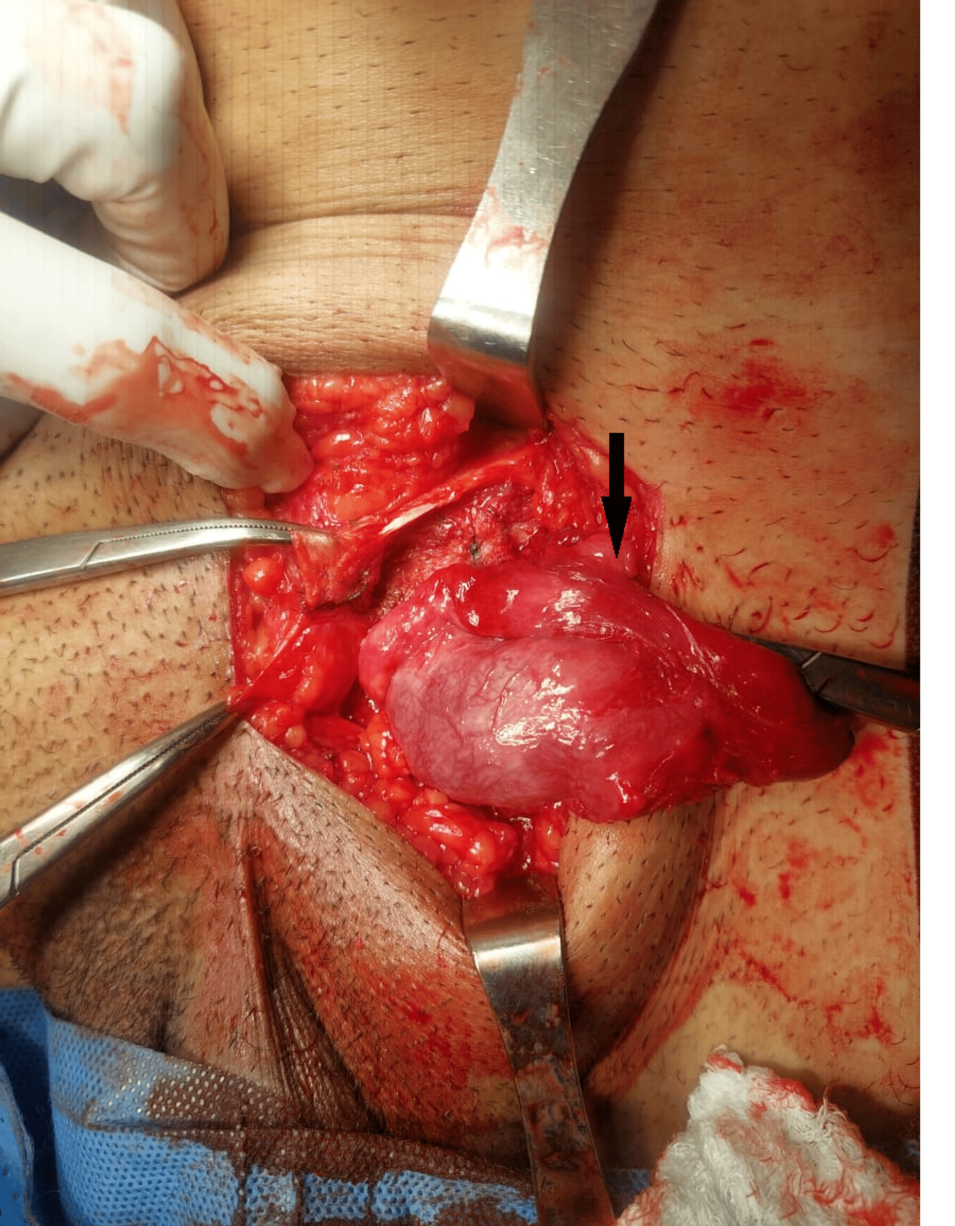
Atrophied left testicle post detorsion The black arrow illustrates the epididymis of the left detorted testicle

Fragments of mesh, along with residual sutures, were identified, and the deep inguinal ring was opened to ensure all incarcerated contents were freed. At this time, it became clear that the mass wedged at the superficial ring was an atrophic testicle. Upon closer inspection of the dusky testicle and cord structures, which were twisted about one another, it was evident that testicular torsion had occurred. Prompt detorsion was performed, resulting in the resolution of the dusky appearance of the organ (Figure 2). 

The on-call urology team was contacted intraoperatively and informed of the findings described above. Urology promptly joined the general surgery team in the operating theatre to confirm findings and decide upon definitive management. After examination, urology determined that the intra-abdominal length of the spermatic cord was insufficient to re-establish repositioning of the now pink, detorted testicle, into the scrotum. Given the lack of spermatic cord length along with the risk of malignant transformation of the ectopic testicle, an on-table decision was made to perform a left orchidectomy. Once orchidectomy was complete, the general surgery team went on to reconstruct the posterior wall of the inguinal canal. The deep ring was completely closed, and a polypropylene mesh was placed to decrease the risk of hernia recurrence. The abdomen was then closed in layers. A histological assessment of the left ectopic testicle showed no malignant transformation, and the patient went on to have a full recovery.

## Discussion

The stage for this patient’s acute presentation had been set years before his emergency presentation to our center. This patient gave a history of blunt trauma to the scrotum in childhood, which had resulted in the disappearance of his left testicle. In his adult life, he had sought answers regarding the missing organ by way of an ultrasound investigation. However, the testicle was never located. The user-dependent nature of ultrasonography cannot be overlooked in this case, and the patient’s verbal report of negative ultrasound findings cannot be taken as solid evidence of complete testicular absence. There was no light shed on the absence of a left testicle upon perusal of the operative notes of the primary hernioplasty. TTD is a rare complication resulting from blunt scrotal trauma [4]. It was first reported by Claubry in 1818, at which time he referred to TTD as traumatic luxation of the testis [5,6]. TTD is characterized by displacement of one or both of the previously normally located testicles outside of the scrotum [7]. This is a rare entity, with less than 200 cases documented in the literature [4], with an incidence of less than 0.5% worldwide [7]. 

TTD is most often unilateral and can be classified based on the location of the dislocation. Internal dislocation occurs when the testicle is forced into the abdomen. Superficial dislocation occurs when the testicle lies outside the abdominal cavity. It is forced subcutaneously within the spermatic cord and can ascend as proximally as the superficial inguinal ring [5]. The condition is seen frequently in motorcycle accidents, and, in such cases, can often be associated with more life-threatening injuries, resulting in a missed diagnosis upon primary and secondary surveys. Although usually a non-fatal complication, if left untreated, TTD can lead to atrophy, necrosis, malignant transformation, and infertility [6].

In the index case, it can be deduced that this patient endured a case of superficial dislocation, resulting in the left testicle lodging proximally within the spermatic cord, but distal to the superficial ring of the inguinal canal. This explains the uneventful left inguinal hernia repair performed 10 years before his acute presentation. Further proximal migration of the testicle, however, may be explained by complications resulting from inguinal hernia repair. Testicular ischemia, atrophy, and necrosis are rare complications that can result from hernia repair involving the use of mesh [2]. However, another noteworthy cause of testicular ischemia is damage to vascular structures supplying the testicle due to excessive dissection of the spermatic cord. Delivery of the testis out of the scrotum and cases which involve large inguino-scrotal hernias also increase the risks of testicular ischemia [1].

The literature documents testicular ischemia resulting in atrophy post-inguinal hernia repair at a rate of 0.2-1.1% [2,8,9]. Despite a lack of consensus on the precise mechanism of infarction within the literature [2], it has been reported that testicular ischemia results from thrombosis of testicular venous drainage via the pampiniform plexus [9,10]. Testicular blood flow is supplied by three main vessels: the testicular, epididymal, and internal spermatic arteries, along with anastomotic networks between them [8]. As a result of the redundancy of arterial supply, testicular ischemia resulting from loss of arterial flow is uncommon. The diagnosis of testicular ischemia is made clinically but can be confirmed via colour or power Doppler ultrasound, which demonstrates no flow within the organs [1]. Wants et al. highlighted the effect of direct contact between the testicular vasculature and mesh, resulting in impairment of testicular blood flow [9]. This effect on blood flow, intuitively, can result in testicular ischemia and atrophy as described above.

Ultimately, the case described involves an acute torsion of an atrophic, dislocated left testicle. Testicular torsion is a urological emergency caused by twisting of the testicular cord and vessels, resulting in acute testicular ischemia [10]. A search of the literature revealed no correlation between the use of mesh in hernia repair and testicular torsion. The authors propose that the sequence of events in this case was as follows: pre-existing superficial TTD was complicated by hernioplasty-induced testicular ischemia, likely secondary to vascular injury due to mesh use or direct vascular trauma. The ischemia resulted in atrophy and shortening of spermatic cord structures, leading to further proximal migration of the already dislocated testicle. The testicle became trapped at the superficial inguinal ring and subsequently underwent torsion.

With the Liechtenstein repair being the most common approach, patients must be counseled on the existing 0.5% risk of testicular ischemia present. Though the incidence of this complication is low, patients must be prepared and well informed, as ischemia and atrophy can lead to grave psychological distress and can affect the quality of life, including the risk of infertility. Tissue repair options remain a valuable approach in patients who have a low risk of hernia recurrence, small indirect inguinal hernias, and those who are not candidates for mesh repair [11]; for example, in cases where infection is involved.

## Conclusions

Ultimately, the type of hernia repair method chosen in these cases is up to the surgeon's preference, comfort level, and experience. However, complications associated with each herniorrhaphy approach must be considered and patients counseled accordingly. While drawing attention to two rare conditions - TTD and testicular ischemia due to the use of mesh - we also aim to highlight the importance of full clinical examination before any surgical intervention. If this patient’s testicular dislocation had been noted upon his first herniorrhaphy 10 years prior, there might have been a chance of testicular salvage.
